# Case Report: Insulin-Dependent Diabetes Mellitus and Diabetic Keto-Acidosis in a Child With COVID-19

**DOI:** 10.3389/fped.2021.628810

**Published:** 2021-02-10

**Authors:** Karin Nielsen-Saines, Erica Li, Adriana Martinez Olivera, Rachel Martin-Blais, Yonca Bulut

**Affiliations:** ^1^Division of Pediatric Infectious Diseases, Department of Pediatrics, David Geffen School of Medicine, University of California, Los Angeles, Los Angeles, CA, United States; ^2^Division of Critical Care Medicine, Department of Pediatrics, David Geffen School of Medicine, University of California, Los Angeles, Los Angeles, CA, United States; ^3^Department of Pediatrics, David Geffen School of Medicine, University of California, Los Angeles, Los Angeles, CA, United States

**Keywords:** COVID-19, children, type 1 diabetes mellitus (T1DM), diabetic keto-acidosis (DKA), SARS CoV-2, pediatric COVID-19

## Abstract

During the COVID pandemic, a surge in pediatric Type 1 Diabetes Mellitus (T1DM) cases appears to be occurring, potentially due to the presence of autoantibody-induced immune dysregulation triggered by COVID-19. We describe one such case in a previously healthy 7-year-old with asymptomatic COVID-19 presenting with a high nasopharyngeal SARS CoV-2 virus load, detectable COVID-19 IgG antibodies, diabetic keto-acidosis and islet cell autoantibodies. COVID-19 is not a trivial disease in children and adolescents and can lead to lifelong sequelae such as T1DM. Raising awareness about a possible association between COVID-19 and T1DM in children is critical.

## Introduction

During the COVID-19 pandemic, the number of cases of T1DM in youth spiked, with evidence suggesting an association between both conditions (1, 2). Studies have long implicated viruses, particularly respiratory infections, as potential triggers of T1DM in children and young adults (3). In a large prospective pediatric study, a temporal association was noted between respiratory infections and development of autoantibodies against insulin-producing pancreatic beta islet cells (3). Following a surge in COVID-19 cases, a prospective registry demonstrated a significant increase in pediatric diabetic ketoacidosis (DKA) diagnoses (1). Between March to May 2020, 532 children in Germany were diagnosed with T1DM, with 45% presenting with DKA. The incidence of DKA in children was nearly double of that reported in the prior year (24.5%) with the risk of DKA in 2020 being 1.85 times higher than in the 2 prior years (2.75 times higher in children <6 years of age as compared to 2019) (1). In the U.K., investigators reported an 80% increase in the number of cases of T1DM in children as compared to prior years (2). The reason for higher rates of DKA in youth could be multi-factorial and related to delayed medical care (4). Findings, however, parallel what was observed in adults with COVID-19 (5).

## Patient Information

A 7-year-old previously healthy Hispanic male with no pre-existing co-morbidities presented to the UCLA emergency department with progressive anorexia and a 10-pound weight loss over 3 weeks in August 2020. Three days prior to presentation, the patient complained of acutely worsened anorexia with polydipsia, abdominal pain, nausea, and headache (Table 1). Both the patient and his mother denied any prior or concurrent presence of fever, cough, nasal congestion, shortness of breath, diarrhea, or dysuria. There was no history of recent illnesses or sick contacts. The child lived in a multigenerational family household in south Los Angeles, including both grandparents who worked as school janitors, his mother who was studying for her degree remotely, a young adolescent cousin and a 13-year-old healthy sister. No one in the household reported recent illnesses and the family history was overall unremarkable.

**Table 1:** Timeline.

Days 1–18	Progressive lack of appetite, gradual weight loss of 10 pounds.
Days 19–21	Acute anorexia, polydypsia, abdominal pain, nausea, and headache.
Day 22 (ED visit and admission to PICU)	Drowsiness, abdominal pain, nausea and headache, anorexia, tachypnea, dry mucous membranes, reduced skin turgor, fatigue, tachycardia, metabolic acidosis on ED visit. POC blood glucose 470 mg/dl. Admitted to Pediatric Critical Care for management. Diagnosis of Diabetic ketoacidosis (DKA) made. Hydration and continuous insulin infusion initiated.
Day 23 (PICU day 1)	SARS CoV-2 nasopharyngeal PCR results are positive; lymphopenia noted in complete blood count; chest X ray showed clear lungs; ECG showing sinus tachycardia and incomplete right bundle branch block. SARS CoV-2 IgG results are positive. Noted to have hypokalemia, hypomagnesemia, hypocalcemia, and hypophosphatemia which are repleted with hydration.
Day 24 (PICU day 2)	Metabolic acidosis and electrolyte imbalance resolved. Continuous insulin infusion switched to a subcutaneous regimen. Repeat SARS CoV-2 nasopharyngeal PCR results are positive.
Day 25–26 (Pediatric Ward)	After adequate glucose control is achieved on subcutaneous regimen, patient is discharged home.

## Clinical Findings

In the emergency department, the patient's initial vital signs were a temperature of 36.8°C, heart rate of 131 beats per minute (BPM), blood pressure of 114/75 mmHg, respiratory rate of 37 breaths per minute and an oxygen saturation of 99% on room air. His weight was 25 kg, height 121.9 cm, and BMI was at the 75% percentile for age. On initial examination, he was drowsy but arousable. His exam was notable for dry mucous membranes, reduced skin turgor, tachypnea, clear lungs clear to auscultation, and soft, non-distended, non-tender abdomen. While he appeared fatigued, he had a non-focal neurological exam and responded appropriately to questions.

## Diagnostic Assessment

An initial point-of-care blood glucose was 470 mg/dL. A complete blood count (CBC) showed a normal white blood cell (WBC) count of 6.69 × 10 E^3^/uL, with an absolute neutrophil count (ANC) of 4,650 cells/uL, as well as a mild lymphopenia (1,420 lymphocytes/μL) (Table 2). The urinalysis was significant for a specific gravity of 1.024, 2+ ketones, 3+ glucose, 2+ protein. Chest X-ray did not show any pulmonary pathology. Clinical and laboratory findings were consistent with diabetic ketoacidosis (DKA). In the emergency department, the patient received a 10 mL/kg normal saline bolus and started on continuous insulin infusion. He was then transferred to the pediatric intensive care unit (PICU) for further care.

**Table 2:** Laboratory values on and during admission with abnormal results highlighted.

**On admission**		
**Parameter**	**Patient value**	**Reference range**
White blood cell count	6.69	5.00–14.50 × 10 E^3^/uL
Hemoglobin	15.4	11.5–15.5 g/dL
Hematocrit	44.0	35.0–45.0%
Platelet count	304	143–398 × 10 E^3^/uL
Neutrophil percent	**69.5**	38–68%
Lymphocyte percent	**21.2**	25–54%
Sodium	130	135–146 mmol/L
Potassium	3.0	3.6–5.3 mmol/L
Chloride	94	96–106 mmol/L
Total CO_2_	**4**	20–30 mmol/L
Anion gap	**32**	8–19 mmol/L
Urea nitrogen	9	9–21 mg/dL
Creatinine	0.47	0.40–0.70 mg/dL
Glucose	**486**	65–99 mg/dL
Calcium	10.1	9.2–10.6 mg/dL
Phosphorus	**2.7**	4.0–5.6 mg/dL
Magnesium	1.8	1.4–1.9 mEq/L
AST	14	≤41 U/L
ALT	<5	≤40 U/L
Glucose, point of care	**470**	65–99 mg/dL
Hemoglobin A1C	**14.8%**	<5.7%
pH, venous	**7.01**	7.31–7.41
pCO_2_, venous	**<15**	40–50 mm Hg
pO_2_, venous	**46**	36–42 mm Hg
Bicarbonate, venous	**3.5**	22–26 meQ/L
Base Deficit, venous	**-28**	−2 to +2
O_2_ Sat, venous	77.2%	60–80%
**Inflammatory markers and COVID-19 laboratory results during admission**	**Detected** **×** **2, average Cts 23.03 and 27.1**	Undetectable
Covid-19 PCR		
Covid-19 IgG	**Detected, OD 29.0**	≤15.0
C-reactive protein	**1.0**	<0.8 mg/dL
Procalcitonin	**0.55**	<0.10 μg/L
Fibrinogen	340	199–409 mg/dL
Ferritin	**412**	8–350 mg/mL
Lactate dehydrogenase	291	<306 U/L
D-Dimer	**1.06**	<0.6 μg/mL
Interleukin-6	<2.0	<2.0 pg/mL
Troponin I	<0.04	<0.1 ng/mL
Brain natriuretic peptide	87	<100 pg/mL
Vit D	**14**	20–50ng/ml
Hbg A1C	**15**	<5.7%
Ig A, serum	**362**	50–240 mg/dL
Transglutaminase Ig A	<20	<20.0 CU
Transglutaminase Ig G	<20	<20.0 CU
ZNT8 (Zinc transporter 8 antibodies)	**140 U/mL**	<15 negative
GAD (Glutamine Acid Decarboxylase Antibody)	**>250 IU/ml**	0.0–5.0 IU/mL
IA-2 (Islet Antigen-2 autoantibody)	**>120**	0.0–7.4 U/mL
Insulin antibody	<0.4	0.0–0.4
C peptide	0.2	1.1–4.3 ng/ml

*Values outside of the reference range is bold*.

On arrival to the PICU, the patient was persistently tachycardic, with heart rates ranging from 100–140 BPM. He required a total of 40 mL/kg of normal saline fluid boluses over 24 h. An electrocardiogram was obtained, which revealed sinus tachycardia with incomplete right bundle branch block. His heart rate improved to 90–100 beats per minute over the next 2 days. A repeat CBC following hydration 24 h later showed a WBC count of 15.27 × 10 E^3^/μL with an ANC of 12,740 cells/μL, persistent lymphopenia (1,430 lymphocytes/μL) and 70 immature granulocytes/μL, hemoglobin of 16.1 g/dL, hematocrit of 46%, platelet count of 362 × 10 E^3^/μL. As the DKA was resolving on the two-bag system, the patient's hypokalemia, hypomagnesemia, hypocalcemia, and hypophosphatemia were repleted as appropriate.

On admission to the hospital, as per current hospital policy, a nasopharyngeal swab was tested for the presence of SARS-CoV-2 RNA using the Thermo Fisher TaqPath assay (Thermo Fisher Scientific, Waltham, MA). This returned positive the next morning, with cycle threshold (Ct) values of 23.25 for ORF1ab target, 23.12 for the N target, and 22.73 for the S target. A repeat upper respiratory specimen obtained 3 days later and evaluated using the BDMax assay (Beckon Dickinson and Company, Franklin Lakes, NJ) also returned positive, with Ct values of 26.7 for the N1 target and 27.5 for the N2 target. LIAISON® SARS-CoV-2 IgG assay targeting the spike protein (S1/S2) (Diasorin S.p.A., Saluggia (VC)—Italy) was also positive on admission (optical density of 29.0; positive ≥ 15.0, range 400 units/mL. The patient never had any fever, diarrhea, nor any respiratory symptoms during the admission. Additional laboratory evaluations for inflammatory markers potentially associated with COVID-19 Multi-Inflammatory Syndrome in Children (MIS-C) are shown in Table 2. Except for very mildly elevated procalcitonin, C-reactive protein, D-Dimer and ferritin levels, inflammatory markers were normal. Markers of type 1 diabetes, however, were grossly abnormal, with an islet antigen 2 (IA-2) autoantibody of >120.0 U/ml and the glutamic acid decarboxylase antibody >250.0 IU/ml. Hemoglobin A1C was elevated at 14.8%.

The patient remained on a continuous insulin infusion and the two-bag system for 2 days and then was switched to a subcutaneous insulin regimen once the acidosis resolved. Adequate glucose control on the new subcutaneous insulin regimen was achieved and he was discharged after a 4-day admission.

## Therapeutic Intervention (Plan of Treatment)

The patient responded well to usual DKA protocol, with somewhat interesting features of high insulin requirements and low potassium. After stabilization of the metabolic acidosis, a subcutaneous insulin regimen was started per endocrinology with the patient transferred to the pediatric ward. He continued on a Lantus insulin regimen for basal coverage with carb correction. A sliding scale with Humolog was initiated. Additional laboratory studies including IgA, IA-2 Ab, insulin Ab, transglutaminase Ab panel, ICA-512-HgbA1c, transglutaminase abs panel GAD 65, anti-insulin antibodies, c-peptide, and Zinc Transporter 8 were ordered (Table 2).

Both the patient and his mother received diabetes education for home regimens. He required electrolyte correction and was discharged home with oral potassium supplementation and 2,000 IU vit D per dietary recommendations. A repeat Covid PCR test was stil positive on 8/24. The patient was discharged home to quarantine with mother for 14 days from the first positive SARS CoV-2 PCR test.

## Follow-up, Expected, and Actual Outcomes

The child was seen in the endocrine clinic 1 day after discharge and again 1 and 3 weeks after discharge, being found to have acceptable blood glucose levels. He was following a carbohydrate-controlled diet fairly well and thus no changes were made during the diabetic nutrition follow-up visits. The expected outcome for new onset T1DM is well-controlled DM care and dietary modification, however, we do not have enough information regarding the long term outcomes of simultaneous T1DM and Covid-19 infection in children.

## Discussion

Our pediatric patient illustrates the typical pattern of T1DM in children during the COVID-19 pandemic and differs from that of a published report in a 19 year old with new onset DKA following COVID-19 (6) as our patient had a classic presentation of autoantibody-mediated T1DM. The timing of SARS-CoV-2 infection in our patient coincided with development of indolent symptoms of diabetes, particularly anorexia and weight loss. Although T1DM is most commonly diagnosed in childhood, it is a relatively rare disease, occurring in about 1.5 in 1,000 children (7). COVID-19 is also infrequently identified in children as compared to adults, with pediatric cases mainly diagnosed during pandemic surges (7). For this reason, the magnitude of the association between T1DM, DKA, and COVID-19 in youth is difficult to quantify, but is, nonetheless, apparent. In a report of U.S. children hospitalized with COVID-19, 2.7% had a history of chronic diabetes, and 2.9% developed DKA during their hospital stay (8). Whether SARS CoV-2 itself or deferred medical care are responsible for a higher presentation of DKA cases in younger populations has been a matter of debate (4, 9).

ACE2 is expressed in multiple organs, including exocrine and endocrine tissues of the pancreas. SARS-CoV, responsible for the epidemic of 2003, was shown to bind to ACE2 receptors through its spike protein, similarly to SARS-CoV-2 (10). Diabetes has been recognized as a risk factor for increased COVID-19 morbidity and mortality in adults since the onset of the SARS-CoV-2 pandemic (11). More recently, data from adults suggest that COVID-19 may lead to worse outcomes in patients with pre-established diabetes, and may trigger diabetic ketoacidosis (12). New-onset diabetes during the course of COVID-19 infection is recognized in both adults and children, with a small number of case reports described to date (2, 13). A study of SARS patients with diabetes strongly suggested that the localization of ACE2 expression in the endocrine part of the pancreas allowed SARS coronavirus to enter and damage pancreatic islets, leading to acute diabetes (10). Both SARS-CoV and SARS-CoV-2 have been reported to trigger transient insulin resistance and hyperglycemia (10, 11).

A report of a 19 year-old male with autoantibody negative Type 1 diabetes mellitus (T1DM) following COVID-19 infection acquired following exposure to symptomatic parents highlights the important consideration of whether SARS-CoV-2 infection may directly damage pancreatic islet cells abundantly expressing ACE2 viral receptors (6). Another possibility is that immune dysregulation during the course of COVID-19 disease may induce development of autoantibodies against pancreatic beta cells. Potentially both circumstances may occur in parallel, with infection of cells expressing ACE2 receptors triggering a dysregulated humoral immune response resulting in the death of pancreatic islet cells. Deferred medical care which discourages patients and parents of children to seek help during lockdown situations may likely contribute to more patients with new onset T1DM present in DKA (9, 14). Decline in pediatric medical care occurs COVID-19 pandemic, where for example childhood immunization programs have suffered despite best efforts (15). In the case of our patient, symptoms went unrecognized for nearly 3 weeks, and upon diagnosis, off-scale levels of autoantibodies were present, a common finding in T1DM. Both mechanisms of pathogenesis and the underlying issues associated with pandemic situations and unavailability of hospital beds are likely leading to an unprecedented number of DKA cases in youth with COVID-19. Table 3 summarizes current pediatric studies on the topic to date.

**Table 3:** Reports of DKA in children and youth during the COVID-19 pandemic.

**Reference**	**Study Findings**
(1)	Comparison of pediatric registry data from diabetic centers in Germany for children and adolescents with newly diagnosed T1DM. The frequency of DKA was significantly higher in 2020 in contrast to the 2 previous years (44.7% in 2020 vs. 24.5% in 2019; aRR, 1.84 [95% CI, 1.54–2.21]; *P* < 0.001; vs. 24.1% in 2018; aRR, 1.85 [95% CI, 1.54–2.24]; *P* < 0.001). The study found a significant increase in diabetic ketoacidosis and severe ketoacidosis at diabetes diagnosis in children and adolescents during the COVID-19 pandemic in Germany.
(2)	Noted an 80% increase in T1DM cases in children during the COVID-19 pandemic as compared to prior years in a cluster of units in North West London, U.K., with evidence of SARS-CoV-2 infection or exposure in a proportion of children tested.
(4)	Incidence of T1DM cases in children Germany did not exceed the expected in 2020, an observation which authors associate with the timing of an effective lockdown which averted SARS-CoV-2 infections.
(6)	Case report of a 19 year old in Germany who presented with autoantibody-negative T1DM after SARS-CoV-2 infection
(8)	Analysis of pediatric COVID-19 hospitalization data from 14 U.S. states between March to July 2020 found that although the cumulative rate of COVID-19–associated hospitalization among children is low compared with adults, one of three hospitalized children are admitted to an intensive care unit. Newly diagnosed DKA identified in 3% of children.
(9)	Cross-sectional study in Italy reporting an increase in DKA presentation among newly diagnosed pediatric patients with T1DM from 36.1% in 2019 to 44.3% in 2020 (*p* = 0.03)
(18)	Case report of a pediatric patient presenting with acute pancreatitis before progressing to multisystem organ dysfunction due to COVID-19 induced MIS-C.

Although SARS CoV-2 infection of pancreatic beta cells has not been yet demonstrated, there is enough evidence of direct viral damage leading to organ failure in different body compartments, as in the case of COVID-19 myocarditis (16). Cases of pancreatitis in patients with COVID-19 have been reported in both adults and children (17, 18). It is difficult to discern which cases of new onset T1DM are due to direct viral damage, and which are due to immune dysregulation induced by COVID-19. While DM is a risk factor for severe COVID-19, SARS-CoV-2 infection also triggers T1DM, a bidirectional relationship shown to occur in adults and now increasingly demonstrable in youth. It is critical that awareness regarding this specific complication of SARS-CoV-2 infection in children be heightened, not only to enable early identification of DM, but also to counteract the belief that COVID-19 poses no threat to young patients.

In summary, our intent with this case report was to raise awareness among pediatricians about the potential for a large increase in the number of COVID-19 associated T1DM and DKA cases in children and youth following pandemic surges. Other institutions might be witnessing the same phenomenon and through this publication we wished to share this unique presentation of COVID-19 in children. Because the number of COVID-19 cases have sky-rocketed in recent months globally (December 2020/ January 2021), it is very likely that we will be seeing a very sharp rise in the number of T1DM and DKA events in children exposed to the virus through their family members. These children often require admission to critical care, and it is very important to recognize this potential complication of this condition in pediatric populations.

## Patient Perspective

Despite the difficult circumstances, the patient and his family are adapting to the diagnosis of T1DM and mother and child are now heavily engaged with our institution's pediatric diabetes clinic. The child continues to be closely monitored with bi-monthly in person and telehealth visits. Family support through pediatric diabetes networks has been instrumental.

## Data Availability Statement

The original contributions presented in the study are included in the article/Supplementary Material, further inquiries can be directed to the corresponding author/s.

## Ethics Statement

The patient's mother provided informed consent for the publication of this case report.

## Author Contributions

All authors listed have made a substantial, direct and intellectual contribution to the work, and approved it for publication.

## Conflict of Interest

The authors declare that the research was conducted in the absence of any commercial or financial relationships that could be construed as a potential conflict of interest.
